# A quantitative criterion for determining the order of magnetic phase transitions using the magnetocaloric effect

**DOI:** 10.1038/s41467-018-05111-w

**Published:** 2018-07-11

**Authors:** Jia Yan Law, Victorino Franco, Luis Miguel Moreno-Ramírez, Alejandro Conde, Dmitriy Y. Karpenkov, Iliya Radulov, Konstantin P. Skokov, Oliver Gutfleisch

**Affiliations:** 1 Dpto. Física de la Materia Condensada ICMSE-CSIC, Universidad de Sevilla, Apdo1065, 41080 Sevilla, Spain; 2 Institut für Materialwissenschaft, Technische Universität Darmstadt, Alarich-Weiss-Str. 16, 64287 Darmstadt, Germany

## Abstract

The ideal magnetocaloric material would lay at the borderline of a first-order and a second-order phase transition. Hence, it is crucial to unambiguously determine the order of phase transitions for both applied magnetocaloric research as well as the characterization of other phase change materials. Although Ehrenfest provided a conceptually simple definition of the order of a phase transition, the known techniques for its determination based on magnetic measurements either provide erroneous results for specific cases or require extensive data analysis that depends on subjective appreciations of qualitative features of the data. Here we report a quantitative fingerprint of first-order thermomagnetic phase transitions: the exponent *n* from field dependence of magnetic entropy change presents a maximum of *n* > 2 only for first-order thermomagnetic phase transitions. This model-independent parameter allows evaluating the order of phase transition without any subjective interpretations, as we show for different types of materials and for the Bean–Rodbell model.

## Abstract (web summary)

Magnetocaloric materials often perform best when their magnetic transitions are at the boundary between first- and second-order behavior. Here the authors propose a simple criterion to determine the order of a transition, which may accelerate future magnetocaloric material searches.

## Introduction

There is a recent increased interest in understanding and unambiguous determining the order of thermomagnetic phase transitions^1–4^, motivated by fundamental studies of the characterization of different phase transitions and due to the important role that phase change materials play in different technological areas, like data storage in rewritable optical memories^5^, thermal energy storage^6^, etc. One timely application of thermomagnetic phase transitions is magnetic refrigeration, whereby a magnetocaloric material acts as a solid refrigerant, replacing the gas compression system employed in refrigerators and air conditioners nowadays^7–10^.

Magnetocaloric materials can be classified into the order of phase transition that they undergo: either first- (FOPT) or second-order (SOPT) type phase transitions. FOPT materials have the advantages of exhibiting large magnetic entropy change and adiabatic temperature change but accompanied by thermal hysteresis, rate dependent behavior and decreased cyclic performance compared to quasistatic single shot characterization as a function of field or temperature. Conversely, SOPT materials do not suffer from thermal hysteresis but their magnetocaloric responses are usually smaller than those of most FOPT materials operating within the same temperature range. Therefore, a current goal of magnetocalorics is to combine the best from both types of materials: large response without hysteresis and a tradeoff between static and cyclic performance^10^. The search for such materials bring forth the intermediate point of FOPT converting to SOPT, which is usually termed tricritical point in literature though the critical point of the SOPT will be more appropriate. LaFeSi-^11,12^ and MnFePSi-type^13^ alloy series are examples exhibiting a gradual change from FOPT to SOPT. From simulations point of view, the most extensively used model to study FOPT materials in magnetocalorics is the Bean and Rodbell model^14^. It reproduces FOPT, SOPT, and a critical point of the SOPT by simply changing a single parameter, *η*^15^: *η* = 0 corresponds to the Brillouin model, $0 \le \eta \,< \,1$ relates to SOPT; *η* > 1 to FOPTs and *η* = 1 ascribes to the critical point of the SOPT.

The formal classification of phase transitions has historically evolved^16^ from the simple conceptual definition given by Ehrenfest^17^ to a modern definition^18^ whereby the change of the order parameter (e.g., magnetization in a ferromagnetic material) across the transition is analyzed. In cases that the order parameter discontinuously changes, the transition corresponds to first-order type while a continuous change implies a higher order or continuous phase transition. At a FOPT, typically a discontinuous change of entropy (Δ*S*) occurs, giving rise to a latent heat at the critical temperature: Δ*Q* = *T*_c_ Δ*S*. In this way, transitions wherein there is no latent heat, like First-Order Magnetization Processes (FOMP)^19,20^, or those where the derivatives of the thermodynamic potential diverge (instead of presenting a discontinuous jump) can be properly classified^16^.

In the magnetocalorics field, techniques for determining the order of phase transitions are distinguished as calorimetric or magnetic. The former consists in measuring the latent heat of the transition^2,21^, whose equipment is highly specific and not easily available in every magnetics laboratory. Moreover, identifying the order of phase transitions using bulk heat capacity alone can become cumbersome in some cases^22^. As a substitute for these calorimeters, purely magnetic techniques have been developed. Among them, the most remarkable one is the broadly used Banerjee criterion^23^, though in some specific cases the resulting interpretations contradict to those arising from calorimetric measurements^3^. This discrepancy arises from the assumption of Banerjee criterion that the material follows a mean field model, which might not apply to all cases. An alternative method was proposed^24^ based on the scaling nature of the magnetocaloric curves of SOPT materials^25–28^. However, controversies regarding the applicability limits of this technique and of scaling itself were raised^15,29^. Furthermore, the method involves extensive calculations (it requires construction of a rescaled curve using different temperature dependent magnetocaloric curves at different applied fields) and the interpretations depend on qualitative data features, which require appropriate data processing and experience. In particular, for cases close to the change of the order of phase transition^4^, the complexity to evaluate the quality of the collapse of the data is highly dependent on subjective interpretations. Just recently, another method based on the Bean and Rodbell model has been reported^30^, but is still based on a particular equation of state that fulfill the mean field approach and, therefore, might not be generally applicable.

A particularly challenging limitation of the existing methods used for identifying the order of phase transitions is the study of composite samples or transition temperature distribution. For SOPT materials, if the distribution of Curie temperatures of their phases are rather separated (typically ~100 K), some of the previously mentioned methods can be successfully applied, sometimes requiring a deconvolution of the data^31^. However, when Curie transitions are nearer, distortions could arise due to either a FOPT or distributed Curie temperatures; as the recently proposed method based on Bean–Rodbell model requires fitting of the data^30^, its applicability is de facto prevented by the distribution of transition temperatures.

In this work, we propose a quantitative method to identify the order of thermomagnetic phase transitions using the field dependence of magnetocaloric effect: for samples with FOPT, Δ*S*_M_ depends on field with exponent *n* > 2. This characteristic fingerprint can be easily identified in a quantitative way. The method does not require rescaled curves construction, interpretations based on overlapping of different curves or data fitting to any specific equation of state. Its applicability is, therefore, not based on any specific magnetization model, making it a generally applicable approach. The method is applied to various systems with different origin of phase transition, namely an alloy series exhibiting FOPT → SOPT based on their compositions (LaFeSi-type), numerical simulations using the Bean and Rodbell model, a Heusler-type alloy and a perovskite cobaltite that exhibits an antiferromagnetic-ferromagnetic FOPT. Furthermore, the applicability to materials with distributed transition temperatures is demonstrated using numerical simulations and experimental composite materials, demonstrating that our proposed method is also valid for heterogeneous, multiphase and composite magnetocaloric materials.

## Results

### La_1_Fe_13−*x*_Si_*x*_-type alloys

La_1_Fe_13−*x*_Si_*x*_-type alloys are selected in this work (*x* = 1.2, 1.4, 1.6, 1.8) to represent alloy series experiencing a change from FOPT to SOPT, which in this case is attained by increasing Si content^4^. They are denoted by their Si content as Si 1.2, Si 1.4, Si 1.6, and Si 1.8 samples. Their order of phase transition will be identified using previously existing techniques (starting with calorimetry and continuing with magnetic measurements) and with our proposed method to enable comparison.

**Fig. 1:**
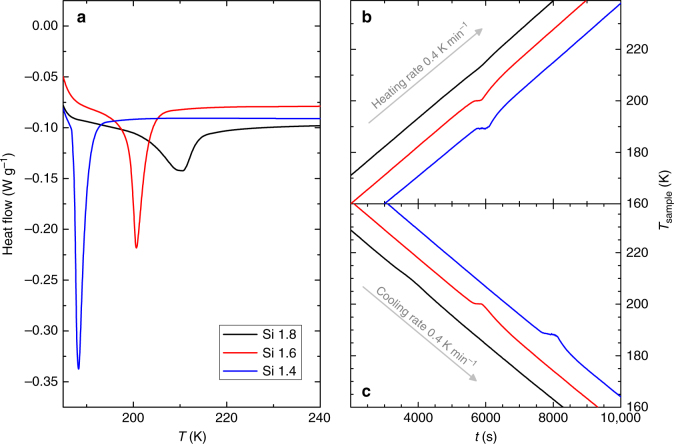
DSC results and time dependence of sample temperatures. **a** Temperature dependence of the heat flow at a constant heating rate of 10 K min^−1^ shows that Si 1.4 and Si 1.6 present FOPT while Si 1.8 undergoes a SOPT; **b** and **c** time (*t*) dependence of the sample temperatures, at a constant heating/cooling rate of sample holder of 0.4 K min^−1^, show a temperature plateau for FOPT samples

Figure 1a shows the temperature dependence of the heat flow measured at a constant heating rate of 10 K min^−1^ using a DSC calorimeter. In the case of FOPT, the curves should show a sharp peak associated to the latent heat, which is observed for Si 1.4 and Si 1.6 samples. Contrastingly, the curve of Si 1.8 shows the characteristics of a SOPT, namely a shallower lambda-like cusp in the heat flow at the transition temperature^32^. The ultimate shape of the curve would be affected by the characteristics of the sample such as disorder, inhomogeneity, etc.^32,33^ The limitation of using DSC to identify the order of the transition is to correctly distinguish the cusp-like feature from a shallow peak and not erroneously attribute a latent heat to the former, whereby overall require much experience of the researcher.

An alternative calorimetric method to identify the order of the transition is to continuously cool/heat the sample while recording its temperature change through the transition to reveal the presence of the latent heat associated with FOPT. While the evolution of sample temperature with SOPT is continuous, for FOPT materials there will be a time interval during which the temperature of the sample should remain constant despite that heat is constantly rejected/absorbed by the sample (a plateau-like behavior). Figure 1b and c shows the presence of these plateaus for Si 1.4 and Si 1.6 under heating and cooling protocols, which gives a clear indication of a FOPT in these two compounds, whereas Si 1.8 exhibits a well-defined SOPT behavior. This is in agreement with the results of the DSC thermograms of Fig. 1a, serving as an additional confirmation of the order of the phase transition of the samples.

**Fig. 2:**
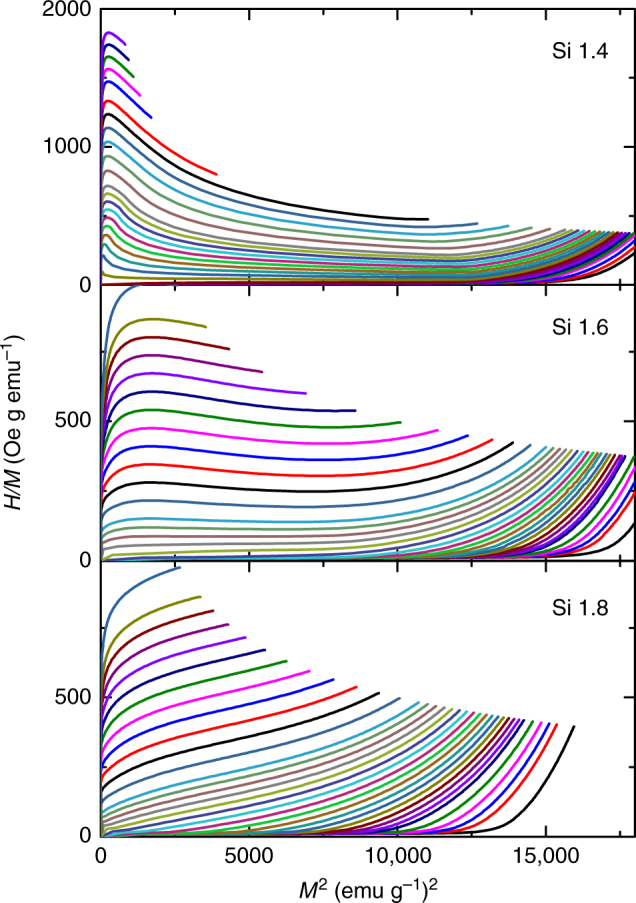
Arrott plots for the Si 1.4, Si 1.6 and Si 1.8 samples. According to Banerjee criterion, both Si 1.4 and Si 1.6 samples exhibit FOPT while Si 1.8 undergoes a SOPT

Arrott plots in combination with Banerjee criterion (Fig. 2) also confirm that samples Si 1.4 and 1.6 are FOPT materials (they exhibit negative slopes) while Si 1.8 undergoes a SOPT (all slopes are positive).

**Fig. 3:**
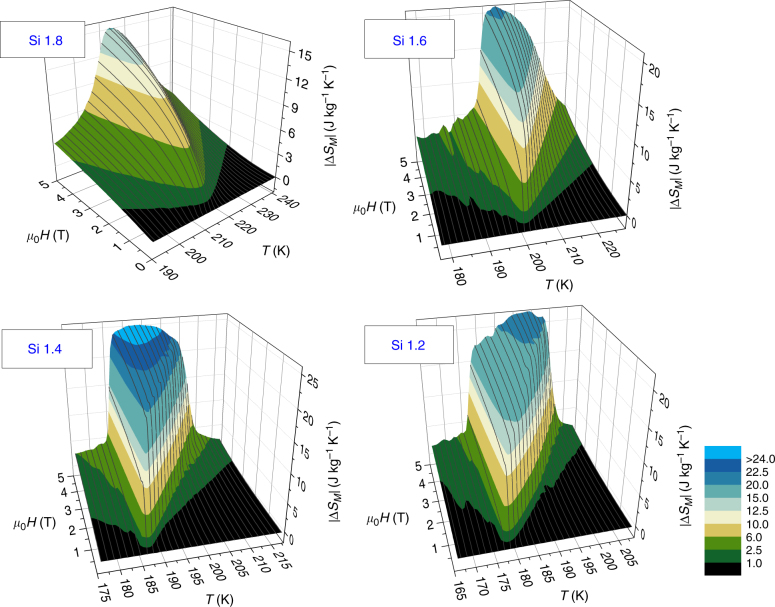
Magnetic entropy change of the studied La_1_Fe_13-*x*_Si_*x*_ alloys. While Si 1.8 exhibits “caret” type behavior, Si 1.4 and Si 1.2 have the shape of a “cliff”. Si 1.6 apparently exhibits one behavior or the other, depending if we look at the low or high temperature parts of the surface

Figure 3 shows the three dimensional plots of the field and temperature dependence of the magnetic entropy change (Δ*S*_*M*_) of the studied alloys. In the case of the SOPT material (Si 1.8), the temperature evolution of Δ*S*_*M*_ is gradual for all magnetic field values. Likewise, there is no abrupt change in the field dependence of this magnitude for any of the isotherms. However, for FOPT materials both their field and temperature dependencies of Δ*S*_*M*_ show an abrupt behavior for certain values of the magnetic field (*H*) or temperature (*T*). It is particularly important to notice that for temperatures above the transition temperature (e.g., for *T* > 185 K in Si 1.4 sample), Δ*S*_*M*_ presents a sharp increase once the field reaches a certain value (which is temperature dependent). The SOPT magnetocaloric curves had been termed as “caret type”^34^; in contrast, we can describe this observation in Si 1.4 sample as a “cliff” in the 3D representation. While this cliff behavior is clearly observed for Si 1.4 and Si 1.2 samples, it is not that evident for Si 1.6, which displays an almost mixed behavior depending if we account for the surface regions of low temperatures or high temperatures. However, both calorimetric and Arrott plot techniques indicated its FOPT character. The ambiguity of the identification of the order of phase transition from the shape of MCE curves is because this composition is close to the critical point of the SOPT (predicted as Si 1.65^4^).

The solution to this problem lays in the cliff shape of the MCE surface and the quantitative description of this feature will further extend its advantages. The lines over the surfaces in Fig. 3 show the field dependence of Δ*S*_*M*_ at selected temperatures for all studied alloys. For Si 1.8 (SOPT), the field dependence behavior is rather similar for temperatures above and below *T*_pk_: a gradual increase of Δ*S*_*M*_ with increasing fields. However, the situation begs to differ for FOPT cases. Looking at the most evident cases (Si 1.4 and Si 1.2), for *T* < *T*_pk_ and while still restricting the observed *T* range close to the transition, there is an abrupt increase of Δ*S*_*M*_, followed by a gradual increase at larger fields. This latter gradual increase corresponds to the apparent plateau above the cliff. For *T* > *T*_pk_, it is seen that, for low fields, Δ*S*_*M*_ has a relatively small value, then abruptly increases to comparatively high values. This sharp increase is temperature dependent and requires increasingly larger fields at higher temperatures after the transition temperature. Although this can be a general trend in the field and temperature dependence of Δ*S*_*M*_ that clearly marks a distinction between FOPT and SOPT materials, this method of identifying the order of the phase transition is still qualitative. The perception of abruptness depends on the appreciation of the researcher and would not be quantitative. Therefore, the results from this method might not be conclusive for the case of Si 1.6.

**Fig. 4:**
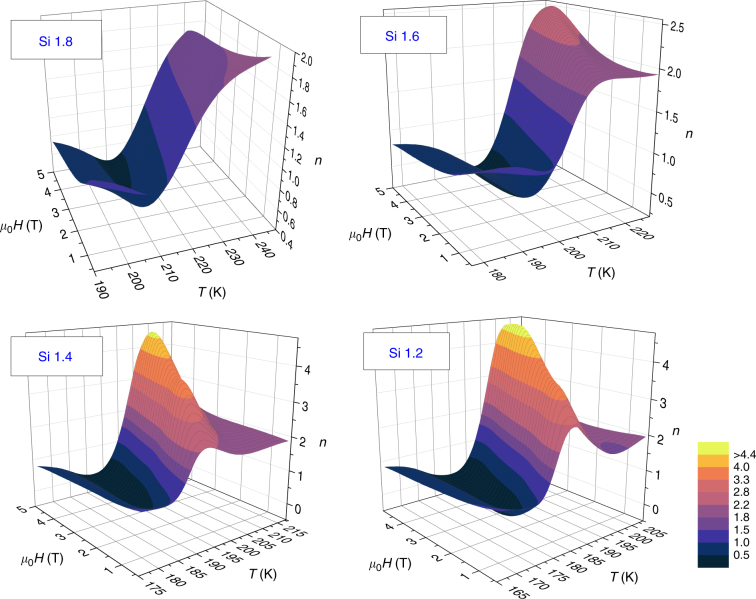
Field and temperature dependence of the exponent *n* for La–Fe–Si alloys. The values of *n* > 2 for Si 1.2, 1.4, and 1.6 clearly indicate their FOPT behavior, while Si 1.8 is a SOPT

To quantify the change in the field dependence, we can use Eq. (4) to calculate the local temperature and field variations of exponent *n* that defines the field dependence of Δ*S*_*M*_. Results are shown as 3D plots in Fig. 4. For very small fields, the sample is in a multi-domain state and, therefore, those values of the exponent should not be considered. The temperature dependence of *n* for SOPT materials was thoroughly studied in the literature^25,35^. At temperatures well below the transition temperature, *n* should have a value that tends to 1; for *T* much larger than *T*_pk_, *n* tends to the paramagnetic value of 2, while for *T*=*T*_pk_ or *T* = *T*_C_ (with *T*_c_ being the Curie temperature)^36^, *n* has a value that depends on the critical exponents of the material ($n = \left( {1 - \alpha } \right)/\Delta = 1 + (1 - 1/\beta )/\delta$), provided that the applied field is in the range that make the material remain within the critical region^15^. This behavior is clearly observed for Si 1.8, where we find a trend of 1→minimum→2 in its *n*(*T*) isofield curves. However, this is dissimilar for the other samples. For these alloys, although the low and high temperature limits (*n* = 1 and *n* = 2, respectively) are still observed, the minimum of *n* is followed by a maximum with a value larger than 2. This maximum is field dependent but is a distinctive feature of FOPT samples: the existence of this overshoot of *n* above 2 is the criterion to determine that the transition is a FOPT. Hence, Fig. 4 clearly indicates that Si 1.6 undergoes a FOPT. A more pronounced maximum of *n* is observed with decreasing Si content, i.e. with a more pronounced FOPT behavior.

### Bean and Rodbell model

To justify that this maximum of *n* as a function of temperature is an intrinsic feature of first-order phase transition materials, we also performed calculations using the Bean and Rodbell model^14^. However, it is important to note that here the currently proposed criterion is not based on the Bean and Rodbell model or connected to the applicability of this model to a specific sample, as we will demonstrate in later sections with experimental results arising from very different families of magnetocaloric materials.

In this paper, parameters similar to those of Gd are used, namely *J* = 7/2, *g* = 2, $N = 3.0\times 10^{28}\,{\mathrm{m}}^{ - 3}$, *λ* = 59 and $\rho = 7.90\,\times 10^3\,{\mathrm{kg}}\, {\mathrm{m}}^{ - 3}$, all with the typical meaning used in the model. Instead of calculating magnetic entropy change from the numerical processing of *M*(*H*,*T*) curves, the discontinuity of magnetization for FOPT cases (i.e. when the *η* parameter of the model is larger than 1) makes it preferable to calculate the magnetic entropy change from the difference of the entropy value at zero field and at field *H*:

1 $${\Delta} S_{M} = S(T,H) - S(T,0)$$

**Fig. 5:**
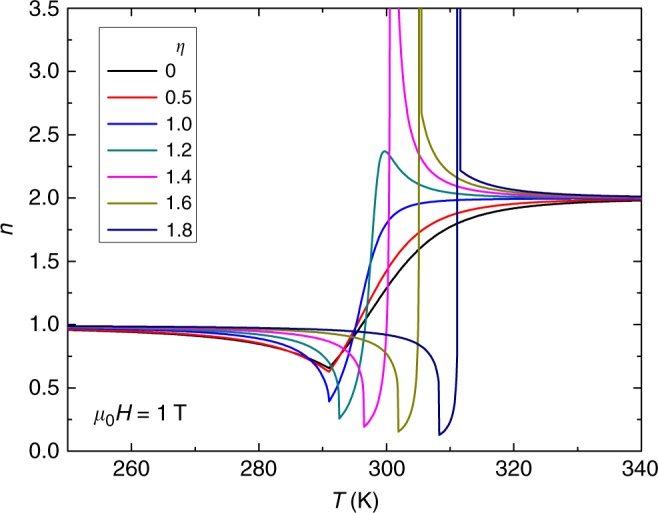
Exponent *n* for the Bean and Rodbell model. Temperature dependence of the field exponent of the magnetic entropy change for cases ranging from SOPT (*η* ≤ 1) to FOPT (*η* > 1) shows that values above 2 correspond to FOPT

**Fig. 6:**
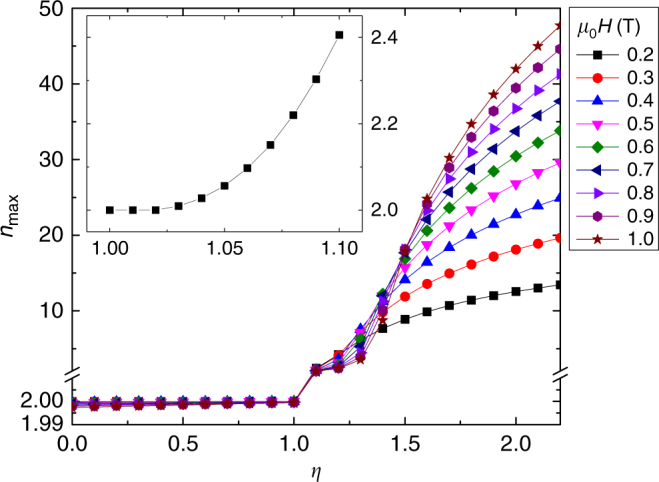
Influence of magnetic field on the maximum exponent *n*. Materials with a FOPT (*η* > 1) exhibit an exponent value larger than 2 for all studied fields. Inset: higher resolution curve for a field of 0.2 T in the proximity of the critical point of the second-order phase transition, where the overshoot of *n* > 2 is already evident for $\eta \ge 1.03$

Once these curves are calculated, the local exponent *n* is determined using Eq. (4). Figure 5 shows the temperature dependence of *n* at a field of 1 T for different values of the parameter *η*, where *η* < 1 corresponds to SOPT, *η* = 1is the critical point of the SOPT and *η* > 1 indicates a FOPT. In agreement with the experimental results, the existence of *n* > 2 after the transition temperature, which subsequently tend to *n* = 2 at higher temperatures, is a distinctive feature of FOPT. As shown in Fig. 6, for each given value of *η*, the maximum value of *n* is field dependent. Likewise, for a given value of the field, *n* increases with increasing *η*. These two features agree with the behavior observed for the La–Fe–Si series with decreasing Si content. The detection of these *n* > 2 values is a quantitative measurement that is not subjectively dependent on the data analysis interpretation or data processing skills of the researcher when attempting to obtain a good collapse of different curves onto a single universal curve. The inset of Fig. 6 shows the proximity of the critical point of the SOPT for an applied field of 0.2 T. As it could be expected, points very close to *η* = 1 exhibit an overshoot that is difficult to detect. However, it is worth mentioning that for *η* = 1.03 the separation from *n* = 2 is already visible by simple inspection. Different models might give different values of the smallest *η* > 1 value for which the overshoot is apparent. Likewise, in the case of experimental samples, the fine details of the phase transition will also dictate when the first order nature of the transition is detectable beyond the experimental noise. This is not a limitation of the currently proposed criterion but intrinsic to any experimental method.

### Heusler alloy

For the case of Ni_45.7_Mn_36.6_In_13.5_Co_4.2_ Heusler alloy, it exhibits low temperature martensite and high temperature austenite, dissimilar to the former La(Fe,Si)_13_ alloys. Its exponent *n* is expected to asymptotically tend to *n* = 2 in the low temperature range and *n* = 1 for high temperatures. There is another major difference with the former La(Fe,Si)_13_ case: the magnetocaloric effect associated to the magneto-structural phase transition is inverse (i.e. $\Delta S_M \,> \,0$) while the high temperature ferromagnetic phase exhibits a small but negative magnetic entropy change (conventional MCE).

**Fig. 7:**
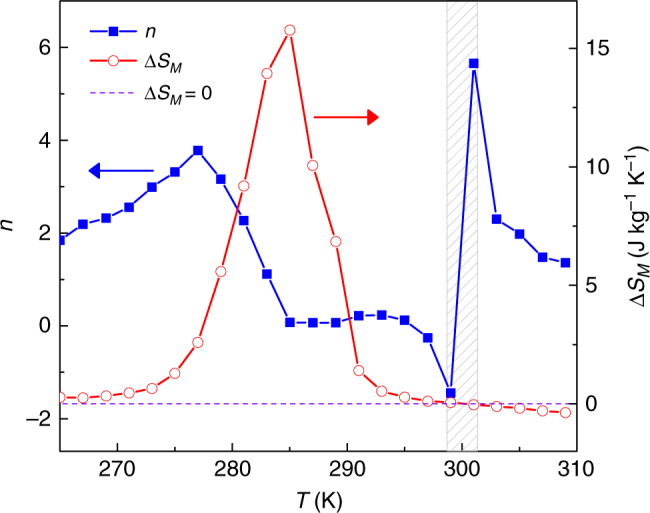
The magnetic entropy change and its field exponent for Heusler-type alloy. The overshoot of exponent *n* occurs near the FOPT of Ni–Mn–In–Co Heusler alloy. Characteristic spikes shown in the shaded gray region indicate the switching of inverse to conventional MCE. The maximum applied field was 1.5 T

Figure 7 shows the temperature dependence of the exponent *n* for the studied Heusler alloy for an applied field of 1.5 T. The main feature that should be observed is the peak of *n* > 2 for a temperature around ~275 K, which corresponds to the martensitic-austenitic phase transition of the first-order type. At ~300 K, there is an anomalous behavior of *n*, which goes below 0 and immediately reaches a spike above 2. This anomaly is related to the change of sign of magnetic entropy change at the transition from inverse to conventional MCE that can be easily identified from the comparison of the temperature dependent Δ*S*_*M*_ and *n* curves. Hence, it does not imply a limitation for the identification of the overshoot associated to a FOPT. It is therefore confirmed that Heusler alloys exhibit the proposed fingerprint of a FOPT, showing the validity of the proposed criterion also for these alloys.

### Composites and inhomogeneous materials

As previously mentioned in the introduction, composite materials are one of the most challenging cases to test the applicability of a method to determine the order of phase transitions due to the presence of a transition temperature distribution arising from inhomogeneities in the sample or due to the presence of several discrete phases. Normally, a deconvolution method is necessary, although that would require the previous knowledge of the phases present in the sample. This severe data processing might then alter the conclusions of the study.

**Fig. 8:**
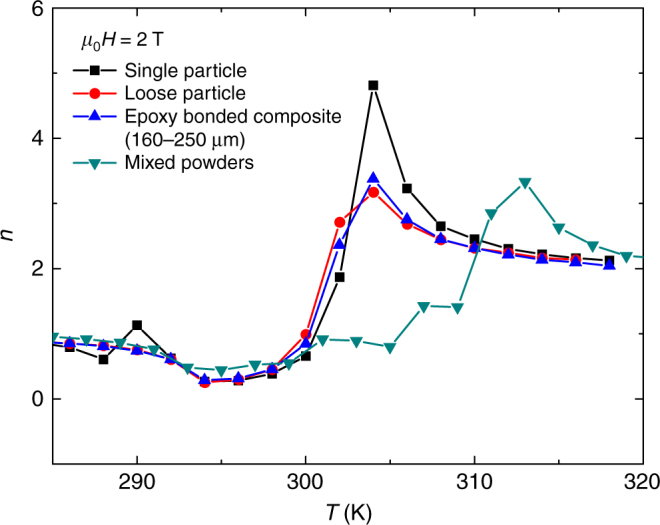
Influence of distributed transition temperatures on exponent *n*. The materials used for this example consist of a single LaFeSi-type particle and different LaFeSi-based composite materials. In all cases, the overshoot of *n* > 2 is observed, in agreement with their FOPT nature

Figure 8 shows the temperature dependence of exponent *n* for a single powder particle of LaFe_11.38_Mn_0.32_Si_1.30_H_1.6_, which exhibits a well-defined transition temperature. In addition, several composite materials (see Methods section for details) are also characterized. The distributed span of the transition temperatures of the composites gives a smoother peak of *n* but clearly with a magnitude larger than 2. Hence, the fingerprint of FOPT is evidently detected and this is observed in all cases of the composite materials. This experimentally demonstrates that inhomogeneities in the material or the presence of different magnetocaloric phases, in principle, do not constitute limitations in this proposed approach to identify the order of phase transition.

**Fig. 9:**
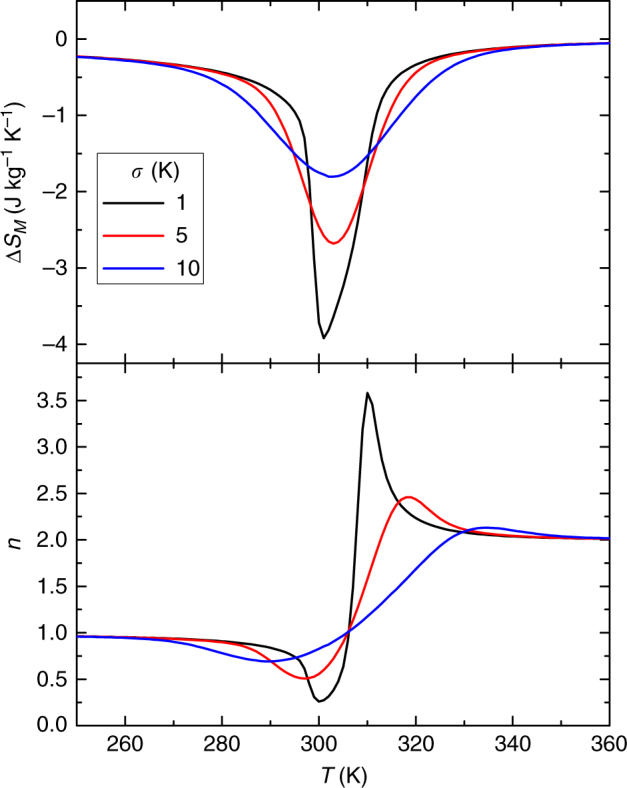
Simulations with a Gaussian distribution of transition temperatures. Three different *σ* values of the Gaussian distribution of transition temperatures in the Bean and Rodbell model for *η* = 1.5 at maximum field of 2.25 T were used. Despite the smooth appearance of the magnetic entropy change (upper panel), which does not resemble FOPT, the overshoot of *n* > 2 near the transition is observed in all cases (lower panel)

From the simulation point of view, a Gaussian distribution of transition temperatures (with 500 discreet values per distribution) has been introduced into the Bean and Rodbell model. Figure 9 shows the magnetic entropy change curves and the temperature dependence of exponent *n* for a maximum field of 2.25 T, *η* = 1.5 and three different values of *σ* of the Gaussian distribution. It is worth noting that even when the magnetic entropy change curves do not resemble a FOPT due to the broad distribution of transition temperatures (*σ* = 10 K) the characteristic overshoot of *n* is still visible. The largest used *σ* of 10 K for a transition close to room temperature corresponds to a full width at half maximum of the distribution of ~24 K, which is larger than that of carefully prepared experimental samples.

### Cobaltite perovskite

**Fig. 10:**
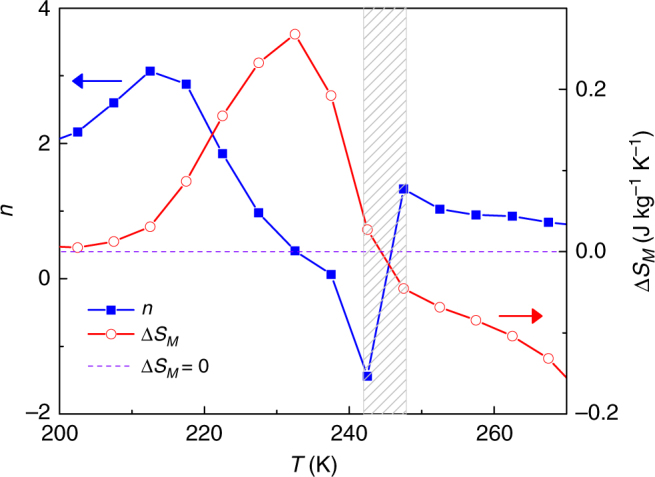
Inverse magnetocaloric response of GdBaCo_2_O_6−δ_ for 1.5 T. The overshoot of *n* is observed near its first order antiferromagnetic–ferromagnetic transition. Characteristic spikes shown in the shaded gray region indicate the switching from inverse to conventional MCE

GdBaCo_2_O_6−δ_ cobaltite perovskite exhibits low temperature FOPT antiferromagnetic-ferromagnetic followed by a ferromagnetic-paramagnetic transition, leading to a narrow ferromagnetic window^37^. The increase of magnetization of the sample upon increasing temperature causes the cooling of the sample upon the application of magnetic field (inverse magnetocaloric effect). This type of transition cannot be described with the Bean and Rodbell model. However, as the criterion we propose here is not based on Bean and Rodbell, it should be capable to describe also this type of transition: The overshoot of *n* > 2 is also clearly observed for the first-order antiferromagnetic-ferromagnetic transition as shown in Fig. 10, underlining the generality of our proposed criterion.

## Discussion

The determination of the order of phase transition of magnetocaloric materials for compositions close to the change from second-order phase transition to first order phase transition is not trivial. Nevertheless, this correct determination is important for fundamental characterization of materials and for studies of their potential application in magnetic refrigerator devices. In this paper we have shown that the field dependence of magnetic entropy change gives a characteristic fingerprint of first-order phase transition materials, which can be used as a quantitative criterion to identify the order of phase transitions: the magnetic entropy change has a dependence that is stronger than a parabolic behavior above the transition temperature for FOPT, which returns to a parabolic behavior at much larger temperatures. This method has been tested on a La–Fe–Si series of alloys with changing behavior from a first order phase transition into a second order phase transition, giving a good agreement with all other known methods based on magnetic measurements for performing this identification. The validity of the proposed procedure has been confirmed using the Bean and Rodbell model, as well as with a Heusler alloy, a set of composite materials and perovskite cobaltites exhibiting an antiferromagnetic-ferromagnetic phase transition.

The main advantage of the method proposed in this paper is that it is quantitative, as opposed to previous methods that rely on different qualitative features of the data, and therefore it does not depend on a subjective choice of a metric to evaluate the appropriate scaling of curves. Two other major limitations of existent methods are also overcome with this fingerprint approach: Unlike other methods based of data fitting, the procedure does not impose a specific equation of state for the material under study, making it general. In addition, it has been shown that this criterion is also valid for multiphase or inhomogeneous materials.

## Methods

### La_1_Fe_13−x_Si_*x*_-type alloys

The La_1_Fe_13−*x*_Si_*x*_ alloy series, with *x* values from 1.2 up to 1.8 was prepared by arc melting, suction casting and subsequent annealing at 1375 K for 12 h. In this compositional range, the order of the phase transition changes from FOPT to SOPT depending on the Si content (from small Si content to large Si content, respectively). The microstructure of the samples was studied by X-ray diffraction and scanning electron microscopy. Results show that the annealed samples contain >98% of La(Fe,Si)_13_ phase. In addition, the results from energy-dispersive X-ray spectroscopy (EDS) show that the actual compositions of the alloys are: La_1.07_Fe_11.2_Si_1.8_, La_1.07_Fe_11.4_Si_1.6_, La_1.12_Fe_11.6_Si_1.4_, and La_1.07_Fe_11.8_Si_1.2_. Hence, the studied samples are denoted by their Si content (*x*). Further details of the magnetic and microstructural characterization are published elsewhere^4^.

### Heusler alloy

A Ni_45.7_Mn_36.6_In_13.5_Co_4.2_ Heusler alloy was prepared by arc-melting and subsequent annealing in a quartz tube at 1173 K for 24 h, under 0.5 bar argon atmosphere, followed by water quenching. ICP-OES confirmed the stoichiometry with a resolution better than 1 at. %. Further details about magnetic and magnetocaloric properties as well as its thermomagnetic hysteresis characterization can be found in the literature^38,39^.

### Composites and inhomogeneous materials

To prepare the magnetocaloric composites, LaFe_11.38_Mn_0.32_Si_1.30_H_1.6_ magnetocaloric powder produced by Vacuumschmelze GmbH had been used. XRD results confirm 95% main phase with NaZn_13_ cubic structure and unit cell with *a* = 11.568 ± 0.002 Å. The initial powder, with a transition temperature *T*_tr_ = 297 K, was sieved and sorted by particles size. Magnetocaloric materials exhibiting a first-order paramagnetic-ferromagnetic transition are very sensitive to changes of their microstructure. At the same time, variation of Mn content by <0.01% can lead to several degrees shift of *T*_tr_. As a result, the *T*_tr_ of different grains of the bulk sample are scattered around *T*_tr_ of the bulk sample. However, the bulk sample exhibits a sharp *T*_tr_ due to the stress-coupling mechanism between the individual grains^40^. The constrain effect is then removed by crushing the bulk sample and the span of the *T*_tr_ of individual fragments is extended in interval of *T*_tr_ ± 3 K, so the loose powder exhibits a distribution of *T*_tr_ that leads to the smoothing of MCE maximum, making this maximum broader but lower. The largest particles (with volume larger than 5 mm^3^) were used as a single particle sample. The loose powder samples were prepared by tight packing them (size 160–250 µm) into weighing paper boxes. Polymer bonded composite samples, in form of pellets or plates, were prepared by mixing magnetocaloric powder with epoxy binder as described in the literature^41^. For the present study, we had chosen composites containing a particle size distribution of 160–250 µm.

To check the consistency of the proposed criterion for a discrete distribution of transition temperatures in a broad temperature range, a final composite sample was prepared by mixing three types of magnetocaloric powders with compositions LaFe_11.83_Mn_0.32_Si_1.28_H_1.6_, LaFe_11.88_Mn_0.3_Si_1.29_H_1.6_, LaFe_11.9_Mn_0.27_Si_1.29_H_1.6_ of *T*_tr_ = 295, 301, and 305 K, respectively. The *T*_tr_ of the particles in this powder composite mixture are extended over a temperature interval of 15 K and can be considered as an example of a non-homogeneous magnetocaloric alloy exhibiting both FOPT and a very broad redistribution of the individual *T*_tr_.

### Cobaltite perovskite

GdBaCo_2_O_6−δ_ samples, provided by Dr. Stevin Pramana, were synthesized via a solid state reaction. Further details about the synthesis and functional properties can be found elsewhere^42^.

### Characterization

Differential scanning calorimetry experiments were performed using a TA-Instruments DSC Q20 with a cooling stage. Measurements were made at a heating rate of 10 K min^−1^ starting with the lowest temperature of the device (185 K). The presence of a time interval at which the temperature of the sample remains constant (i.e., another characteristic feature of FOPT) was measured in a custom made magnetocaloric rig developed at TU-Darmstadt. In this case, the time dependence of the temperature of the sample holder is carefully adjusted using a PID controller to adopt a slow temperature ramp (0.4 K min^−1^). The temperature of the sample is independently measured using a different thermocouple. The slow ramp rate is necessary to avoid the influence of the heat capacity on the evolution of the sample temperature. Faster ramp rates or a sizeable increase of the sample mass would lead to an undesirable lag in the sample temperature.

Magnetization measurements were performed using a Vibrating Sample Magnetometer by recording isothermal magnetization vs. magnetic field curves, *M*(*H*), in a discontinuous protocol that involved the following steps for LaFeSi type alloys: (a) the sample was pre-heated in zero field to a temperature value well above its transition temperature; (b) then cooled down in zero field to the measuring temperature; and (c) measured with increasing field up to the maximum applied field. The field dependence of the transition temperature justifies the specific choice of the protocol: the transition temperature increases with field for LaFeSi-type materials. However, the behavior is different for the Heusler alloy: it decreases with field. This prompts for a modification of the measurement protocol for the latter: step (a) was modified to cooling the sample in zero field to a temperature value well below its transition temperature; followed by (b) heating in zero field up to the measuring temperature. Step (c) remained unmodified. Samples were shaped as thin plates (3 mm wide and 0.1 mm thick) and magnetized with the field in plane, to minimize the possible effect of demagnetizing factor. As the temperature region of the first order phase transition implies the coexistence of magnetic and non-magnetic phases, the correction of the demagnetizing field cannot be accurately performed and any correction would be an approximation. However, the effect of the demagnetizing field close to the transition is small and does not produce any overshoot of the exponent *n*.

Δ*S*_*M*_ was indirectly determined from magnetization measurements up to 5 T using Maxwell relation (where the initial magnetic field is zero):

2 $$\Delta S_M = \mu _0{\int\nolimits_0^H} {\frac{{\partial M}}{{\partial T}}{\mathrm{d}}H' }$$

As indicated in the literature, Δ*S*_*M*_ curves calculated using this discontinuous protocol will exhibit no spurious results regardless of the order of the phase transition^43,44^.

The field dependence of the magnetic entropy change is represented as a power law of the field

3 $$\Delta S_M \propto H^n$$

with an exponent *n* that, in general, is field and temperature dependent. It can be locally calculated as^45^:

4 $$n(T,H) = \frac{{{\mathrm{d}}\ln \left| {\Delta S_M} \right|}}{{{\mathrm{d}}\ln H}}$$

### Data availability

The data that support the findings of this study are available from the corresponding author upon reasonable request.
